# A novel clinical prediction model for hip fractures: a development and validation study in the total population of Sweden

**DOI:** 10.1016/j.eclinm.2024.102877

**Published:** 2024-10-05

**Authors:** Peter Nordström, Viktor H. Ahlqvist, Marcel Ballin, Anna Nordström

**Affiliations:** a Department of Public Health and Caring Sciences, Clinical Geriatrics, Uppsala University, Uppsala, Sweden; b Department of Biomedicine, Aarhus University, Aarhus, Denmark; c Institute of Environmental Medicine, Karolinska Institutet, Stockholm, Sweden; d Centre for Epidemiology and Community Medicine, Region Stockholm, Stockholm, Sweden; e Department of Medical Sciences, Rehabilitation Medicine, Uppsala University, Uppsala, Sweden; f School of Sport Sciences, UiT, The Arctic University of Norway, Tromsø, Norway

**Keywords:** Prediction model, Hip fractures, Bone specific treatment, Guidelines

## Abstract

**Background:**

Low bone density and osteoporosis are indications for bone-specific treatment. However, given the limited availability of bone density data in clinical practice and the fact that most patients with hip fracture do not have osteoporosis, accurate prediction of hip fracture risk in the absence of bone density data would be crucial.

**Methods:**

This development and validation study included the entire Swedish population aged 50 years or older in 2005 (N = 3,340,977) and was conducted by cross-linking data from nationwide registers. Potential predictive variables included diagnoses, prescription medications, familial factors, frailty-related factors, and socioeconomic factors. The primary endpoint was the 5-year risk of hip fracture. Fracture prediction algorithms were developed and validated using multivariable models. Model performance and validation was also examined in a sub cohort restricted to 504,431 individuals with non-Swedish background.

**Findings:**

During a total follow-up of 15.2 million person-years, 87,089 individuals suffered a hip fracture within 5 years. In the final prediction model, 19 variables were associated with a population attributable fraction of 93.9% (95% CI, 93.7–94.1) in women and 92.7% (95% CI, 92.2–93.0) in men. The strongest predictor, besides old age, was the use of homemaker service, with a 5-year risk of hip fracture of 7.8% in women and 4.7% in men. The diagnoses most strongly predicting the 5-year risk of hip fracture was Parkinson’s disease (6.8% in women, 4.6% in men) and dementia (6.1% in women, 3.6% in men). Validation of the prediction model suggested that the optimal threshold for treatment with bone-specific agents was an estimated 5-year hip fracture risk of 3%. Assuming a threshold of 3% and a 30% relative risk reduction from bone-specific treatment, the number needed to treat to prevent one hip fracture was estimated to 36 in women and 52 in men. Similar results were obtained in the sub cohort with non-Swedish background.

**Interpretation:**

A clinical prediction model developed and validated in the total Swedish population could predict the risk of hip fractures with high precision even in absence of data on bone density. The model was associated with a population attributable fraction for hip fracture of more than 90%, and the strongest predictor besides old age was the use of homemaker service, which likely reflect frailty. Based on the model, individuals with an estimated 5-year risk of hip fracture of at least 3% could be considered for bone-specific treatment.

**Funding:**

None.

**Research in context**

**Evidence before this study**

We searched standard databases such as PubMed, and used search engines such as Google, to identify relevant literature written in English until September, 2024, using key words such as “hip fractures”, “prediction”, “prediction tools”, “risk factors”, “FRAX, “algorithms”, “bone specific treatment”, and “guidelines”. Hip fractures are associated with a significant morbidity and decreased self-dependency, with 25% of patients dying within the first year of the fracture. Prevention is therefore of high importance. A few fracture algorithms have been constructed to identify individuals at high risk and guide initiation of bone-specific treatment. However, the most used algorithm, FRAX, only include a selected set of diagnoses, although many other risk factors for fractures are known. In addition, there is lack of validation of the threshold suitable for bone-specific treatment based on any of the existing fracture risk algorithms, and there is also lack of algorithms based on nationwide data that have been shown predict hip fractures with high accuracy without access to bone density.

**Added value of this study**

In this study including the total population of Sweden above 50 years of age, the strongest predictor of hip fracture, besides old age, was the use of homemaker service, with a 5-year risk of hip fracture of 7.8% in women and 4.7% in men. The final prediction model included 19 variables that was associated with a population attributable fraction of above 90% in both women and men. Validation of the final prediction model suggested that the optimal threshold for treatment with bone-specific agents was an estimated 5-year hip fracture risk of 3%. Assuming this threshold to initiate treatment and a 30% relative risk reduction from bone-specific treatment, the number needed to treat to prevent one hip fracture was estimated to 36 in women and 52 in men.

**Implications of all the available evidence**

The risk of hip fractures can be predicted with high precision even in absence of data on bone density. Validation of the current prediction model suggested that a threshold 3% would be associated with a number needed to treat below 60 in both men and women.

## Introduction

Hip fractures in an ageing population present a significant and escalating public health challenge, marked by well-documented morbidity and mortality.^1^^,^^2^ This severity is emphasized by a 30-day mortality rate that can reach up to 10%, escalating further to 25% within the year following the fracture.^3^ In addition, the post-fracture quality-of-life for most surviving patients is notably compromised.^4^^,^^5^ Given that 14 million hip fractures occur globally each year,^6^ they also impose a substantial burden on healthcare and welfare services,^2^ which may increase with increased life expectancy in the future.

Measured bone mineral density and osteoporosis are important indications for bone-specific treatment. However, because of a global shortage of diagnostic equipment, and because only a minority of patients experiencing a fracture have osteoporosis,^7^^,^^8^ there is need for algorithms that can accurately estimate fracture risk, irrespectively of osteoporosis and without measurements of bone mineral density. Currently, FRAX is the most used algorithm to predict fractures worldwide,^9^ although the regression estimates for the different predictors used in the algorithm are not presented. Therefore, additional validated fracture risk algorithms based on nationwide data sources could be of value.^10^ In addition, there is lack of validation of the threshold suitable for bone-specific treatment based on any of the existing fracture risk algorithms.

The primary aim of this study was therefore to develop and validate a clinical prediction model for the 5-year risk of hip fracture, which could be used routinely in clinical practice without the need to measure bone density, among all individuals aged 50 years or older in Sweden. A secondary objective was to evaluate the optimal fracture risk threshold for treatment initiation.

## Methods

### Study design

This study was an observational study conducted through cross-linkage of data from Swedish nationwide registers. Linkage of data across registers were conducted using the Personal Identification Number (PIN), which is unique to everyone living in Sweden.^11^ The study was approved by the Swedish Ethical Review Authority (Number 2013 86/31). Informed consent was waived given that all data were obtained from registers. The methods and results are reported according to the Transparent Reporting of a multivariable prediction model for Individual Prognosis or Diagnosis (TRIPOD) guidelines.^12^

### Derivation of study cohort

The study cohort included all individuals aged 50 years or older and that resided in Sweden on 31 December 2005, with a recorded PIN and sex in the Total Population Register (N = 3,340,977).^13^ In a validation sub cohort, the cohort was restricted to individuals with non-Swedish background, defined as individuals that were either born outside Sweden, or individuals where both parents were born outside Sweden. The non-Swedish background criteria was chosen with the intention of including non-random variation in predictors, outcome, and model performance. This resulted in a total of 504,431 individuals included in the sub cohort, corresponding to 15.1% of the primary cohort.

### Selections of predictors

We collected information on potential predictors in terms of individual-level data on diagnoses, prescription medications, indirect measures of frailty, familial factors, and socioeconomic variables, from different nationwide registers. From the National Patient Register,^14^ we collected data on diagnoses using International Classification of Diseases (ICD) codes, version 10 (Table 1). Data was available for inpatient care since 1997, and for outpatient specialist care since 2001. The diagnoses and medication were selected based on their prevalence in the population and on their associations with fractures in previous studies.^15^ Diagnoses recorded in the National Patient Register has been validated with positive predicted values of 85%–95% for most diagnoses.^14^ Fractures were identified using ICD-10 codes S12–S82, excluding S62. In the National Patient Register, the positive predictive values for fractures in general range between 70% and 87%, with higher values for hip fractures.^14^^,^^16^, ^17^, ^18^ The look-back period for previous fracture was 8 years. Falls in individuals not seeking specialized hospital care are not captured in the National Patient Register, and could not be included in the analyses. Falls severe enough to result in seeking hospital care (although not fractures), are likely set with lower accuracy in the registers, and were therefore not included in the analyses. From the Prescribed Drug Register, we collected information on prescription medications using Anatomical Therapeutic Classification codes. This register includes data on all expedited drugs at pharmacies in Sweden since July 2005. From the Social Service Register, we obtained information about use of homemaker service and residence in a long-term care facility in 2007 and later.^19^ From the Longitudinal Integrated Database for Health Insurance and Labour Market Studies,^20^ we collected data on disposable income in 2005, and from the Total Population Register we collected data on country of birth.^13^ Finally, to investigate the role of familial liability in predicting the risk of hip fracture, we identified all full siblings in the population using data from the Multi-Generation Register.^13^

**Table 1:** Baseline characteristics of the cohort including all men and women at least 50 years of age and living in Sweden 2005 (N = 3,340,977).

Variable	Women (N = 1,764,140)		Men (N = 1,576,837)	
Age, years ± SD	67.3 ± 11.6		65.1 ± 10.5	
Income, Euro ± SD^a^	12,925 ± 19,492		18,194 ± 86,023	
Born in Sweden, N %	1,496,158	84.8	1,340,388	85.0
Hip fracture in full sibling, N %	2865	0.1	2788	0.1
Fracture in full sibling, N %	25,421	0.7	25,369	0.8
Long-term care facility resident, N %^b^	67,967	3.9	29,796	1.9
Homemaker service, N %^b^	154,091	8.7	72,343	4.6
**Diagnoses at baseline, ICD-10 code, N %**				
Alcohol abuse, F10	10,726	0.3	27,072	0.9
Angina pectoris, I20	72,690	2.1	103,283	3.2
Any fracture, S12–S82, excluding S62	183,584	5.2	81,582	2.6
Asthma, J45	31,677	0.9	19,001	0.6
Atrial fibrillation, I48	65,324	1.9	77,555	2.5
Bipolar disease, F31	4730	0.1	3185	0.1
Cancer, C	268,881	7.6	156,126	4.9
Crohn’s disease, K50	5376	0.2	4521	0.1
Chronic obstructive pulmonary disease, J44	26,125	0.7	23,456	0.7
Colitis, K51	7525	0.2	8294	0.3
Dementia, F00, F01, F03, G30	32,847	0.9	23,300	0.7
Diabetes mellitus, E10, E11	118,423	3.4	141,797	4.5
Myocardial infarction, I21	39,331	1.1	68,633	2.2
Osteoporosis, M81	18,899	0.5	2423	0.1
Parkinson's disease, G20	6271	0.2	7302	0.2
Renal disease, N17–N19	2945	0.1	4340	0.1
Rheumatoid arthritis, M05, M06	27,324	0.8	10,450	0.3
Stroke, I61, I63, I64	51,457	1.5	55,028	1.7
Thyrotoxicosis, E05	15,461	0.4	3141	0.1
Traumatic brain injury, S06	44,954	1.3	60,904	1.9
**Medication at baseline,**^c^**ATC-code, N %**				
Antidepressants or depression, N06A	249,939	7.1	116,319	3.7
Bisphosphonates, M05BA	56,371	1.6	6896	0.2
Glucocorticoids, H02AB06	85,264	2.4	50,829	1.6
Levothyroxine, H03AA01	182,356	5.2	32,649	1.0
Neuroleptics or psychosis, N05A	53,151	1.5	32,267	1.0
Sedatives, N05C	296,059	8.4	140,441	4.4

a Data was missing for a total of 1164 individuals for income.

b People living in a long-term care facility or having homemaker service 2007 and earlier.

c Medication at baseline is defined as at least one dispensed dose between 1 July 2005 and 31 December 2005.

### Outcome

The primary outcome of the models was a hip fracture within 5 years after baseline. The outcome of hip fracture was selected, instead of any major fracture since hip fracture have the most severe consequences with respect to morbidity and mortality.^3^ Hip fractures were identified using the National Patient Register and the ICD-10 code S72. To avoid double-counting of hip fractures in individuals who sought care for their historical fractures, we only counted main diagnoses, and did not count incident hip fractures recorded within 12 months of a previous hip fracture. The positive predictive value for a hip fracture as recorded in the National Patient Register is 95%–98.4%.^14^

### Ethics statement

The research adhered to the guidelines set forth by the World Medical Association's Declaration of Helsinki and received ethical approval from the local Ethics Committee and the Swedish Ethical Review Authority (Number 2013-86-31 with amendments).

### Statistical analysis

Descriptive results are presented as means, medians, standard deviations, and quartiles. In all models, individuals with bisphosphonate treatment at baseline (Table 1) were excluded since the aim was to predict fracture risk to guide initiation of treatment with bone-specific agents in individuals naïve to treatment. Follow-up time was restricted to 5 years for the primary analysis. Thus, all individuals were followed from 31st of December 2005 until date of hip fracture, date of death, or 5 years of follow-up, whichever came first. In a Supplementary analysis, results are presented for 10 years of follow-up. In a first model including all putative predictors, hazard ratios (HRs) and predicted risks (1-Survival) were estimated using flexible parametric survival models with baseline knots placed at the 25th, 50th, and 75th percentile of the uncensored log survival times.^21^ Predictors with an independent HR of at least 1.2 in men or in women were considered in later models (Table 2), together with age and income. In addition, an interaction term was added between age and all predictors. The final model included the 19 predictors that were associated with the highest independent predicted risk of hip fracture. The estimated individual hip fracture risk based on these 19 predictors, were further evaluated using hip fracture thresholds from 1 to 10%, for specificity, sensitivity, Receiver Operating Curve Area, proportion of correctly classified individuals (%), and number needed to treat (NNT) to avoid one hip fracture for different thresholds, with a hypothetical treatment effect of 30% based on previous randomised trials.^22^^,^^23^ In addition, the population attributable fraction (PAF), attributed to the independent effects of all predictors combined in the final model was estimated using the *punafcc* command. The PAF provides estimates of potential impact of a risk factor on the occurrence of a given outcome and is calculated based on the prevalence of the exposure and the strength of the association between the exposure and the outcome. We did not assess the temporal validity of the models. Data was missing for 1164 individuals for income. Only complete cases were analysed. All analyses were conducted in Stata version 16 MP.

**Table 2:** Independent risk factors for 5-year risk of hip fracture.

Variable	Women (N = 1,707,769)		Men (N = 1,569,941)	
	HR	95% CI	HR	95% CI
Age, per year-increase	1.10	1.10–1.10	1.10	1.10–1.10
Income, per €1000-increase	0.93	0.92–0.94	0.89	0.87–0.90
Born in Sweden	1.24	1.20–1.28	1.28	1.22–1.34
Hip fracture in full sibling	1.77	1.33–2.36	1.31	0.92–1.86
Fracture in full sibling	0.91	0.79–1.03	1.15	1.00–1.33
Long-term care facility resident	1.10	1.08–1.13	1.24	1.19–1.29
Homemaker service	1.62	1.59–1.65	1.84	1.78–1.90
**Diagnoses and medications**				
Alcohol abuse	2.62	2.40–2.87	2.46	2.30–2.62
Angina pectoris	1.02	0.99–1.06	0.97	0.93–1.01
Any fracture	1.40	1.37–1.43	1.96	1.89–2.02
Asthma	1.00	0.95–1.06	0.94	0.86–1.03
Atrial fibrillation	1.10	1.07–1.14	1.14	1.10–1.19
Bipolar disease	0.99	0.85–1.16	1.04	0.84–1.30
Cancer	1.08	1.05–1.10	1.22	1.18–1.25
Crohn’s disease	1.44	1.24–1.67	1.34	1.08–1.67
Chronic obstructive pulmonary disease	1.57	1.49–1.65	1.52	1.42–1.61
Colitis	0.95	0.83–1.10	1.07	0.90–1.26
Dementia	1.64	1.59–1.70	1.85	1.76–1.94
Diabetes mellitus	1.25	1.22–1.28	1.22	1.18–1.27
Myocardial infarction	1.06	1.02–1.10	1.02	0.98–1.07
Osteoporosis	1.37	1.28–1.45	2.07	1.71–2.50
Parkinson’s disease	2.11	1.96–2.27	2.51	2.32–2.72
Renal disease	1.15	1.00–1.33	1.50	1.31–1.72
Rheumatoid arthritis	1.41	1.33–1.50	1.49	1.34–1.67
Stroke	1.15	1.11–1.18	1.33	1.28–1.39
Thyrotoxicosis	1.10	1.01–1.19	1.18	0.95–1.47
Traumatic brain injury	1.10	1.06–1.15	1.34	1.28–1.40
Antidepressants or depression	1.19	1.16–1.21	1.30	1.25–1.34
Glucocorticoids	1.20	1.16–1.24	1.17	1.10–1.23
Levothyroxine	1.01	0.99–1.04	1.00	0.94–1.07
Neuroleptics or psychosis	1.23	1.19–1.27	1.35	1.27–1.43
Sedatives	1.08	1.06–1.10	1.16	1.13–1.20

CI = confidence interval. HR = hazard ratio.

### Role of the funding source

There was no specific funding for the present study.

## Results

### Study cohorts

Baseline characteristics of the two study cohorts are presented for women and men separately in Table 1. About 85% of the population had a Swedish background. The most common diagnoses at baseline were cancer, previous fracture, and diabetes mellitus, whereas sedatives and antidepressants were the most common prescription medications.

### Predictors of fractures

During follow-up, a total of 87,089 individuals (2.6%) suffered a hip fracture within 5 years (representing 26.8% of all fractures), across a total follow-up time of 15.2 million person-years. The mean age at hip fracture was 82.4 years (range 50.3–109.1). In the first model including all putative predictors, the eight strongest predictors for hip fracture among women included age (HR, 1.10, 95% CI, 1.10–1.10, per year-increase), income (HR, 0.93, 95% CI, 0.92–0.94, per €1000-increase), alcohol abuse (HR, 2.62, 95% CI, 2.40–2.87), Parkinson’s disease (HR, 2.11, 95% 1.96–2.27), hip fracture in a sibling (HR, 1.77, 95% CI, 1.33–2.36), dementia (HR, 1.64, 95% CI, 1.59–1.70), use of homemaker service (HR, 1.62, 95% CI, 1.59–1.65), and chronic obstructive pulmonary disease (HR, 1.57, 95% CI, 1.49–1.65) (Table 2). In men, the eight strongest predictors were similar, but included a previous fracture (HR, 1.96, 95% CI, 1.89–2.02) instead of a hip fracture in a sibling (HR, 1.31, 95% CI, 0.92–1.86) (Table 2). The corresponding results for the 10-year risk of hip fracture is shown in Supplementary Table S1.

**Fig. 1:**
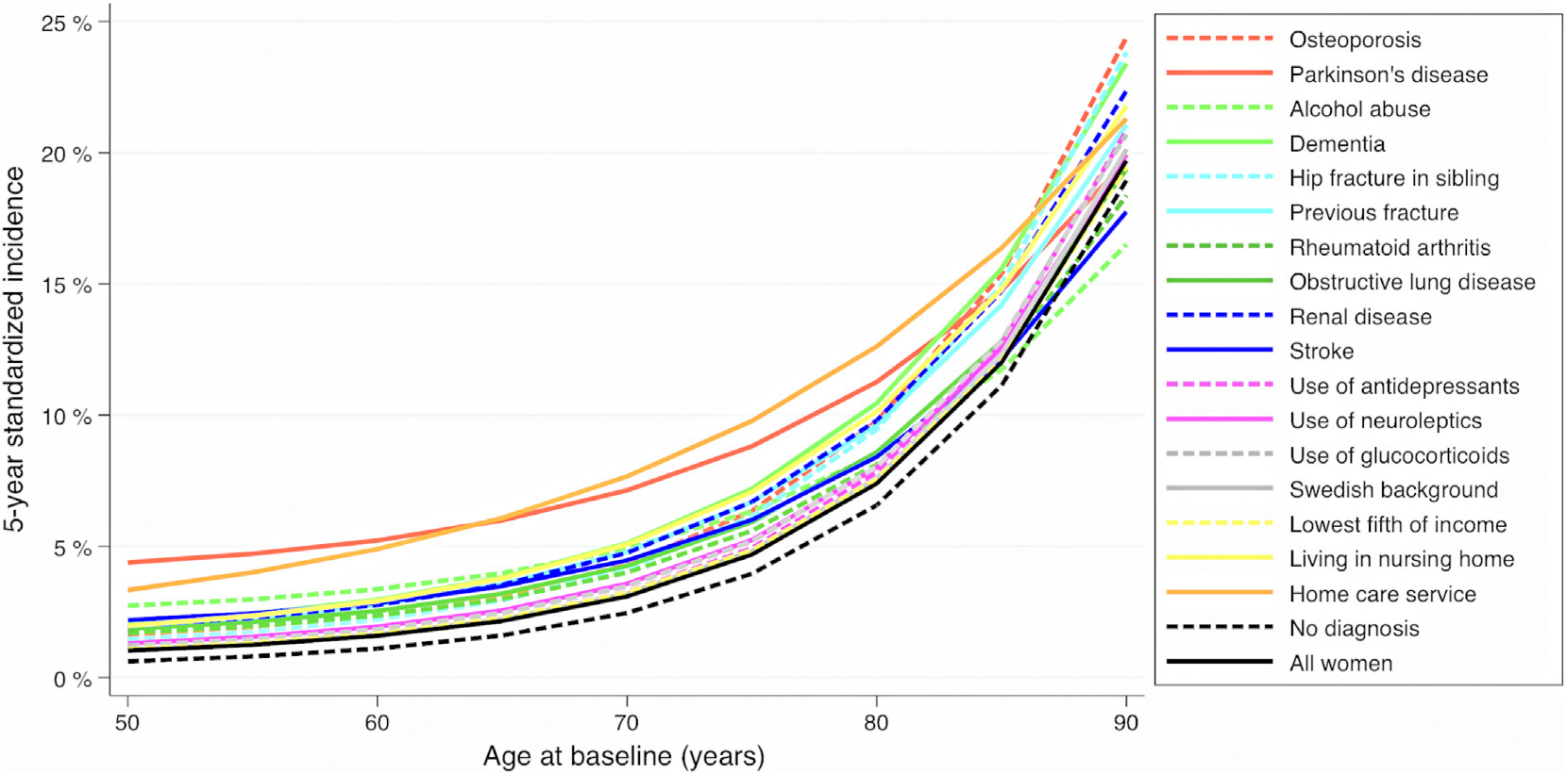
**Standardized individual 5-year risk of hip fracture according to age at baseline in all women and by different risk factors.** Estimates were obtained using flexible parametric survival models with baseline knots placed at the 25th, 50th, and 75th percentile of the uncensored log survival times.

**Fig. 2:**
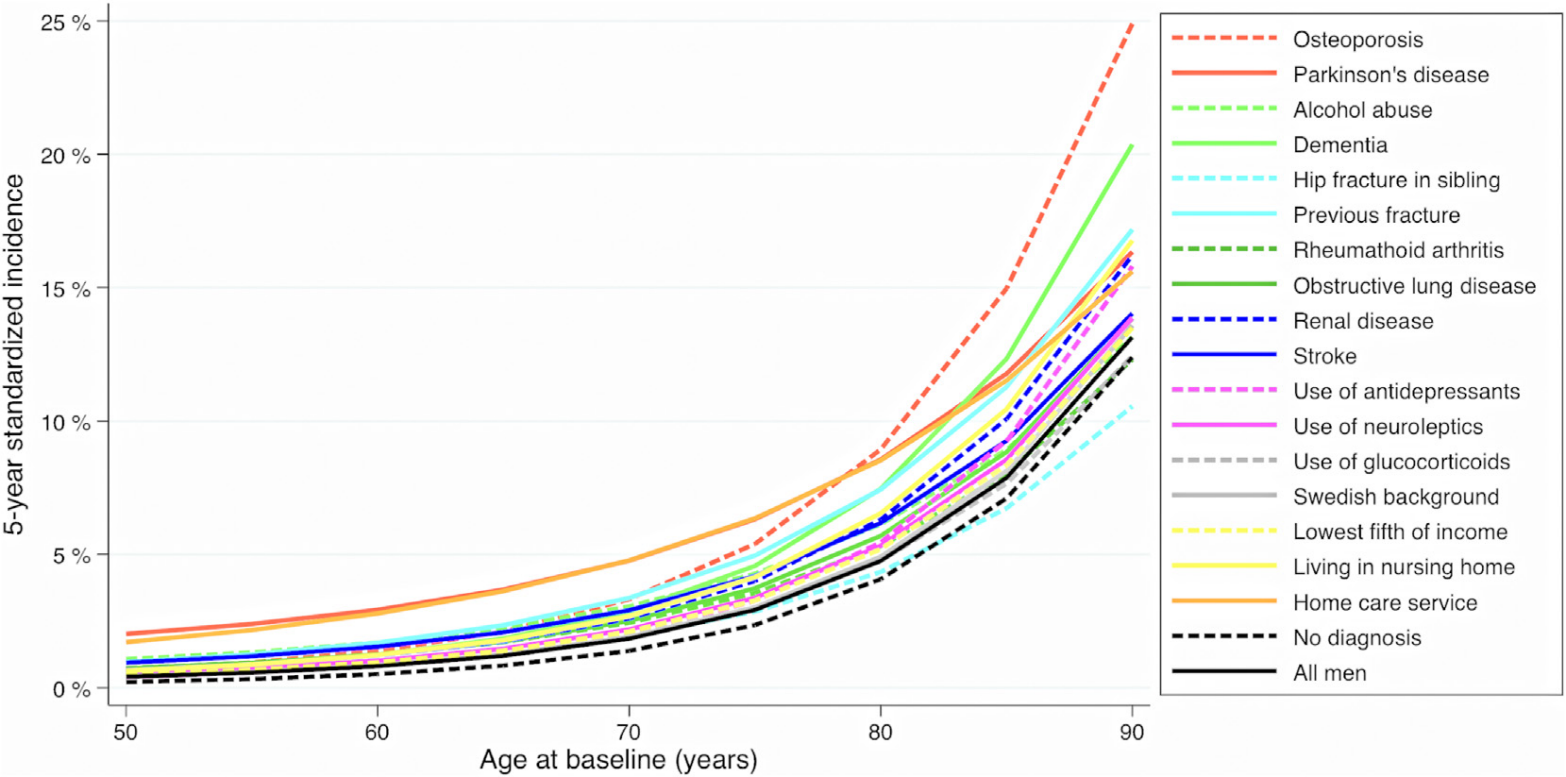
**Standardized individual 5-year risk of hip fracture according to age at baseline in all men and by different risk factors.** Estimates were obtained using flexible parametric survival models with baseline knots placed at the 25th, 50th, and 75th percentile of the uncensored log survival times.

The final model included 19 predictors, where the mean predicted 5-year risk for hip fracture was estimated to 4.4% in women and 2.3% in men (Table 3). Overall, use of homemaker service was associated with the highest risk of hip fracture, where the 5-year risk of hip fracture was 7.8% and 4.7%, in women and men respectively. Furthermore, the role of predictors varied by age in both women (Fig. 1) and men (Fig. 2). Collectively, the 19 predictors were associated with an aggregated PAF for the 5-year risk of hip fracture of 93.9% (95% CI, 93.7–94.1) in women, and 92.7% (95% CI, 93.2–93.0) in men (Supplementary Table S2). The role of the different predictors during a maximum of 10 years of follow-up is shown in Supplementary Fig. S1 in women and Supplementary Fig. S2 in men, with similar results as for 5 years of follow-up. The PAF for the 10-year risk of hip fractures was 91.7% (95% CI, 91.5–91.9) in women and 90.7% (95% CI, 90.4–91.0) in men. The predicted hip fracture risk for each of the 19 predictors in the final model is shown in Supplementary Table S3.

**Table 3:** Predicted individual 5-year risk of hip fracture in all women and men and by selected diagnoses, living conditions, medications, and income at baseline.

Variable	Women				Men			
	Mean %	Median %	25th percentile	75th percentile	Mean %	Median %	25th percentile	75th percentile
Osteoporosis	5.8	1.6	0.5	6.6	4.3	1.0	0.4	4.0
Parkinson’s disease	6.8	3.4	1.5	9.0	4.6	2.1	0.9	5.5
Alcohol abuse	5.2	2.1	0.8	6.6	3.2	1.2	0.5	3.5
Dementia	6.1	2.1	0.8	7.5	3.6	0.9	0.3	3.5
Hip fracture in sibling	5.6	1.5	0.5	6.4	2.2	0.7	0.3	2.2
Previous fracture	5.6	2.1	0.8	6.9	3.7	1.3	0.5	4.0
Rheumatoid arthritis	5.1	1.7	0.6	6.1	2.9	0.9	0.3	2.9
Chronic obstructive disease	5.8	1.9	0.7	6.9	3.1	0.9	0.3	3.1
Renal disease	5.0	1.9	0.7	6.1	3.2	1.1	0.4	3.4
Stroke	4.8	1.6	0.6	5.7	2.7	0.9	0.4	2.8
Depression or antidepressants	4.7	1.2	0.4	5.1	2.7	0.6	0.2	2.4
Psychosis or neuroleptics	4.8	1.3	0.5	5.4	2.6	0.7	0.3	2.6
Swedish background	4.5	1.2	0.4	5.0	2.4	0.6	0.2	2.3
Lowest fifth of income	4.5	1.2	0.4	4.9	2.5	0.8	0.3	2.4
Long-term care facility resident	5.9	2.3	0.9	7.6	3.2	0.9	0.4	3.3
Homemaker service	7.8	5.0	2.4	11.1	4.7	2.5	1.2	6.1
Oral corticosteroids	4.8	1.3	0.4	5.3	2.4	0.6	0.2	2.3
No diagnosis or drugs	3.9	0.9	0.3	4.1	1.9	0.4	0.2	1.7
All individuals	4.4	1.1	0.4	4.8	2.3	0.6	0.2	2.2

The estimated risks are independent and also adjusted for the influence of age.

The results of the final model were validated as sensitivity, specificity, % correctly classified, ROC-area, and the number needed to treat (NNT) with bone-specific agents to avoid one hip fracture using different fracture thresholds (Table 4). The sensitivity was higher in women and the specificity was higher in men. The ROC-area was maximized at a hip fracture threshold of about 3% in both women and men. With an assumed effect of 30% from bone-specific treatment, the NNT in women decreased from 53 individuals with a hip fracture threshold of 1% down to 24 individuals with a fracture threshold of 10% (Table 4). In men, the NNT decreased from 82 to 31 for the corresponding fracture thresholds (Table 4). The corresponding estimates for 10 years of follow up are presented in Supplementary Table S4, where the ROC-area was maximized at a higher threshold. In addition, the observed compared to the estimated 5-year risk of hip fracture was evaluated using calibration slopes in women (Supplementary Fig. S3) and men (Supplementary Fig. S4). For women, the concordance was strong for observed hip fracture risks up to 10%, while for men, this applied up to 4%. For observed higher risks of hip fracture, the estimated risk tended to be overestimated.

**Table 4:** Validation of different thresholds for the predicted individual 5-year risk of hip fracture in the total population using the final prediction model.

Threshold %	Women					Men				
	Sensitivity	Specificity	Correctly classified%	ROC area	NNT	Sensitivity	Specificity	Correctly classified%	ROC area	NNT
1	94.7	49.5	51.0	72.1	52.8	88.6	62.0	62.5	75.3	82.3
2	90.0	62.7	63.7	76.4	41.8	80.5	74.5	74.6	77.5	61.6
3	85.7	69.5	70.1	77.6	36.3	73.4	80.7	80.6	77.1	51.6
4	81.5	74.1	74.4	77.8	32.8	66.8	84.7	84.4	75.8	45.4
5	77.7	77.4	77.4	77.5	30.3	60.6	87.5	87.0	74.1	41.3
6	74.1	79.9	79.7	77.0	28.5	55.1	89.6	89.0	72.4	38.1
7	70.4	81.9	81.5	76.2	27.1	49.9	91.2	90.5	70.6	35.6
8	66.8	83.7	83.1	75.2	26.0	45.1	92.5	91.7	68.8	33.8
9	63.4	85.1	84.3	74.3	25.2	40.9	93.6	92.6	67.2	32.3
10	60.1	86.3	85.4	73.2	24.4	36.8	94.5	93.4	65.6	31.1

The model included the 19 predictors presented in Supplementary Table S2.

NNT = number needed to treat to prevent one hip fracture assuming a treatment effect of 30%.

The final prediction model was evaluated in all individuals with non-Swedish background (N = 504,431). Descriptive characteristics are presented in Supplementary Table S5. During a total follow up time of 2.4 million years, 7241 individuals suffered a hip fracture at a mean age of 78.6 (range 50.2–106.8) years. As in the total cohort, the strongest risk factors for hip fractures included age, use of homemaker service, Parkinson’s disease, dementia, alcohol abuse, and chronic obstructive pulmonary disease (Supplementary Table S6). The overall 5-year risk for hip fracture was estimated to 2.7% in women and 1.1% in men (Supplementary Table S7). As in the total cohort, the importance of the different risk factors varied with age in both women (Supplementary Fig. S5) and men (Supplementary Fig. S6). For all 17 risk factors combined (hip fracture in sibling was excluded because of the poor linkage to relatives in those with non-Swedish background), the aggregate PAF for hip fracture was 90.4% (95% CI, 89.6–91.1%) for women and 85.0% (95% CI, 83.8–86.0%) for men. In this cohort, the ROC-area was maximized at a hip fracture threshold of about 2% in women and 1% in men (Supplementary Table S8).

## Discussion

This study identified several important predictors that were associated with high risk of hip fracture in the total Swedish population. Among these predictors, age, use of homemaker service and Parkinson’s disease were associated with the highest risk for hip fracture. Overall, the final model included 19 predictors that were associated with a PAF for hip fracture well above 90% in both men and women. Evaluation of the model suggested an optimal fit for a hip fracture-risk-threshold of 3% in both women and men. With such a threshold for treatment with bone-specific agents, we estimated NNT during five years to avoid one hip fracture to 36 in women and 52 in men. The importance of the predictors used in the final model were confirmed in a sub cohort including only individuals with a non-Swedish background.

Given the lack of equipment to measure bone density, and the fact that most individuals with fractures do not have osteoporosis, it would be important if fracture risk could be determined without measuring bone density. The 19 predictors included in the final model accounted for a PAF for hip fracture of 93.9% among women and 92.7% among men, suggesting that the model explains almost the full variation in population hip fracture rates. It is also if interest that the strongest identified predictors for hip fracture are not included in the FRAX algorithm, currently most often used in clinical practice.^24^^,^^25^ Some if these predictors included the use of homemaker service, Parkinson’s disease, dementia, and chronic obstructive pulmonary disease. In contrast, most of our predictors are included in the Qfracture algorithm,^10^ where the individual risk of fracture is estimated from a wide range of predictors. Importantly, in our final prediction model, all predictors in women and half in men were associated with a predicted hip fracture risk of more than 3%. Thus, especially women with these risk factors should be considered for bone specific treatment.

It would be of high importance to determine the optimal threshold for treatment with bone-specific agents. Several parameters would influence this threshold including the incidence pattern of hip fractures with increasing age, effects and side effects of bone-specific agents, recommended length of treatment before evaluation, and the influence of different thresholds on the ability to predict hip fractures. Based on a maximized ROC-area and the other factors mentioned above, a threshold for treatment of 3% could be suggested both in women and men in the Swedish population based on the results of the present study. With this threshold, the average woman and man in Sweden would pass a hip fracture risk of 3% in Sweden at 71 years for women and 75 years for men. With an average age at hip fracture of 82 years, assessment at this age would be adequate for the general population but should be earlier for individuals with the risk factors listed, and irrespectively of age e.g., for individuals using homemaker service or individuals with Parkinson’s disease. The suggested threshold for treatment of 3% should be set into perspective. Most guidelines suggest a threshold for treatment of 3% for hip fracture, but importantly during a follow up time of 10 years.^26^ Our algorithm with 10 years of follow up estimates that the average Swedish women would reach 3% hip fracture risk at 63 years of age, i.e., about 20 years before the mean age when hip fracture occurs. With a recommended treatment time of no more than 5 years before evaluation, this may not be optimal. Irrespectively, there seems to be limited support for the thresholds used in current guidelines. For example, a previous report that reviewed 120 guidelines concluded that no rationale was given for the suggested fracture thresholds other than that this was the threshold used by the National Osteoporosis Foundation of the US.^26^ We estimated that a fracture threshold of 3% was associated with a NNT below 60 with an assumed relative effect of bone-specific treatment of 30%.^22^ With the cost for bisphosphonate treatment of less than £100 for five years, the cost to avoid one hip fracture would be less than £6000. In comparison, a recent study estimated that the costs associated with hospital stays alone amounted to £14,462 in the first year for patients with hip fracture.^2^ Given that these fractures also result in increased cost from sheltered living and homemaker service,^27^ apart from personal suffering and death,^28^ bone-specific treatment in accordance with the present hip fracture algorithm also has the potential to be highly cost-effective.

Regardless of fracture threshold to recommend treatment it seems necessary that any fracture algorithms are evaluated in different populations. In the present study, we evaluated the results in the part of the population with non-Swedish background. The same predictors were found to be of importance for hip fracture, but the hip fracture risk was lower, and the mean age of hip fractures was lower. Yet, based on the estimated ROC-area for the different thresholds, and that 3% hip fracture risk occurred at a mean age of 73 years in women and 75 years in men, a threshold of 3% for treatment could hypothetically also be used also in individuals with non-Swedish background. The fact that the estimated PAF was a little lower in this cohort in both men and women is likely related to the fact that the background information registered in national registers could be less accurate due to recent immigration. Thus, it would be preferably if the present hip fracture algorithm could be evaluated also in other large nationwide cohorts.

There are some limitations of the present study that should be considered. First, access to bone density data would have been of value to investigate the importance of this risk factor when accounting for the other variables included in the final risk prediction model. It seems likely that many of the considered risk factors may capture some of the risk conferred by measured bone mineral density. Second, given the number of predictors used, there is the risk of overfitting. This would mean that the algorithm would not produce accurate risk of hip fracture in other populations. Although we could validate the model in the sub cohort of individuals with non-Swedish background, validation in other nationwide cohorts would be of great value to construct country-specific algorithms and thresholds for treatment where possible, and to validate the predictors of hip fracture found in the present study. Third, as with any prediction model we have made simplifying assumptions, such as the absence of complex interactions and non-linear effects. However, even with these simplifying assumptions the total PAF of the developed model seems largely saturated. An important strength of this study is that we included the entire population of individuals living in Sweden, aged 50 years or older. By cross-linking data from several nationwide registers with high quality we were able to assess the role of many potential predictors. Another strength is the focus on a model with 5 years of follow up, which increased the predictive ability compared to the model with 10 years of follow up. The use of nationwide registers also meant that with we had virtually no loss to follow-up. The validation of the results in the sub cohort with non-Swedish background and a lower fracture risk, suggest that the identified predictors are valid also in other countries.

In summary, we developed and validated a clinical prediction model involving 19 parameters that predicted the 5-year risk of hip fracture with a PAF above 90% in both men and women. The most important predictor besides age was the use of homemaker service, which likely reflects frailty. In addition, several diagnoses were identified where the estimated high risk of hip fracture suggest that bone-specific treatment should be considered in both men and women. Based on validation of the model, a threshold for bone-specific treatment of 3% could be suggested for clinical practice. With such a threshold for treatment, the NNT to avoid one hip fracture was below 60 for both men and women in the total cohort, and thus, also highly cost effective. The hip fracture algorithm based on the results from the present study, is free for clinical use and a website is under construction to host the algorithm (www.healthy-ageing.life).

## Contributors

PN and AN conceived the study. All authors designed the study. PN acquired the ethical permission and the data. PN performed the statistical analyses. PN and VHA verified the underlying data. All authors interpreted the data. PN and AN drafted the manuscript. All authors critically revised the manuscript for intellectual content. All authors gave final approval of the version to be published. All authors had full access to all the data and had final responsibility for the decision to submit for publication.

## Data sharing statement

The data files used for the present study are publicly unavailable according to regulations under Swedish law.

## Declaration of interests

We declare no competing interests.
