# Supplementary material for: A novel clinical prediction model for hip fractures: a development and validation study in the total population of Sweden
Source: eClinicalMedicine. 2024 Oct 5;77:102877. doi: 10.1016/j.eclinm.2024.102877 (PMC11490797; doi:10.1016/j.eclinm.2024.102877)
Supplement: Supplementary Figs. S1–S6 and Tables S1–S8 [file mmc1.docx]

**Supplementary material to:**

**A novel clinical prediction model for hip fractures: a development and validation study in the total population of Sweden**

Peter Nordström, MD, PhD^1#^, Viktor H Ahlqvist, PhD^1,2,3^, Marcel Ballin, PhD^1,4^, Anna Nordström, MD, PhD^5,6^

1Department of Public Health and Caring Sciences, Clinical Geriatrics, Uppsala University, Uppsala, Sweden. ^2^Department of Biomedicine, Aarhus University, Aarhus, Denmark.

3Institute of Environmental Medicine, Karolinska Institutet, Stockholm, Sweden. ^4^Centre for Epidemiology and Community Medicine, Region Stockholm, Stockholm Sweden.

5Department of Medical Science, Rehabilitation Medicine, Uppsala University, Uppsala, Sweden. ^6^School of Sport Sciences, UiT The Arctic University of Norway, Tromsø, Norway. #Corresponding author

**Table of Contents**

Supplementary table 1. Independent risk factors for hip fracture during 10 years of follow up 3

Supplementary table 2. Population attributable fraction (PAF) for the different risk factors and in total

for the outcome of hip fracture during 5 years of follow up……………………………………………………4

Supplementary table 3. Predicted individual 10-year risk of hip fracture in all individuals and by selected diagnoses, living conditions, medications, and income at baseline 5

Supplementary table 4. Validation of different thresholds for the predicted individual 10-year risk of hip fracture in the total population using the final prediction model 6

Supplementary table 5. Descriptive characteristics of the cohort with non-Swedish background 7

Supplementary table 6. Independent risk factors for 5-year risk of hip fractures in individuals with non- Swedish background 8

Supplementary table 7. Predicted individual 5-year risk of hip fracture in all 497 399 individuals with non-Swedish background and by selected diagnoses, living conditions, medications, and income at baseline……………………………...………………………………….……………………………….........9

Supplementary table 8. Validation of different thresholds for the predicted individual 5-year risk of hip fracture using the final prediction model in individuals with non-Swedish background…………………...10

Supplementary figure 1. Predicted individual 10-year risk of hip fracture according to age at baseline in all women and by different risk factors………………………………………………………………………...11

Supplementary figure 2. Predicted individual 10-year risk of hip fracture according to age at baseline in all men and by different risk factors 12

Supplementary figure 3. Observed compared to estimated risk of hip fracture using calibration slopes in women during 5 years of follow up……………………………………………………………………………..13

Supplementary figure 4. Observed compared to estimated risk of hip fracture using calibration slopes in men during 5 years of follow up………………………………………………………………………………...14

Supplementary figure 5. Predicted individual 10-year risk of hip fracture according to age at baseline in all women with non-Swedish background and by different risk factors 15

Supplementary figure 6. Predicted individual 10-year risk of hip fracture according to age at baseline in all men with non-Swedish background and by different risk factors 16

Supplementary table 1. Independent risk factors for hip fracture during 10 years of follow up.

| **Women**  **(N=1 707 769)** | | | **Men**  **(N=1 569 941)** | |
| --- | --- | --- | --- | --- |
| **Variable** | HR | 95% CI | HR | 95% CI |
| Age per year to increase | 1·11 | 1·11 to 1·11 | 1·11 | 1·11 to 1·11 |
| Income per €1000 to increase | 0·93 | 0·92 to 0·94 | 0·89 | 0·88 to 0·90 |
| Born in Sweden | 1·25 | 1·23 to 1·28 | 1·30 | 1·26 to 1·34 |
| Hip fracture in full sibling | 1·53 | 1·26 to 1·84 | 1·36 | 1·08 to 1·72 |
| Fracture in full sibling | 1·00 | 0·92 to 1·08 | 1·10 | 1·00 to 1·21 |
| Nursing home resident | 1·01 | 0·99 to 1·03 | 1·12 | 1·08 to 1·17 |
| Homemaker service | 1·39 | 1·36 to 1·41 | 1·68 | 1·64 to 1·73 |
| **Diagnoses and medications** |  |  |  |  |
| Alcohol abuse | 2·50 | 2·33 to 2·68 | 2·42 | 2·30 to 2·54 |
| Angina pectoris | 1·02 | 1·00 to 1·05 | 0·97 | 0·94 to 1·00 |
| Any fracture | 1·38 | 1·36 to 1·40 | 1·91 | 1·86 to 1·96 |
| Asthma | 1·02 | 0·98 to 1·06 | 0·94 | 0·87 to 1·00 |
| Atrial fibrillation | 1·11 | 1·08 to 1·13 | 1·15 | 1·11 to 1·18 |
| Bipolar disease | 1·12 | 1·00 to 1·25 | 1·03 | 0·87 to 1·21 |
| Cancer | 1·07 | 1·05 to 1·08 | 1·20 | 1·17 to 1·22 |
| Crohn’s disease | 1·40 | 1·25 to 1·56 | 1·31 | 1·12 to 1·54 |
| Chronic obstructive pulmonary disease | 1·51 | 1·45 to 1·57 | 1·55 | 1·47 to 1·63 |
| Colitis | 0·96 | 0·86 to 1·06 | 1·07 | 0·95 to 1·21 |
| Dementia | 1·62 | 1·57 to 1·67 | 1·80 | 1·73 to 1·88 |
| Diabetes mellitus | 1·32 | 1·29 to 1·34 | 1·26 | 1·23 to 1·29 |
| Myocardial infarction | 1·07 | 1·04 to 1·11 | 1·02 | 0·99 to 1·06 |
| Osteoporosis | 1·33 | 1·27 to 1·40 | 1·97 | 1·67 to 2·31 |
| Parkinson's disease | 2·27 | 2·13 to 2·41 | 2·87 | 2·69 to 3·06 |
| Renal disease | 1·19 | 1·06 to 1·33 | 1·46 | 1·30 to 1·63 |
| Rheumatoid arthritis | 1·44 | 1·37 to 1·50 | 1·46 | 1·35 to 1·59 |
| Stroke | 1·16 | 1·13 to 1·19 | 1·36 | 1·32 to 1·41 |
| Thyrotoxicosis | 1·13 | 1·06 to 1·20 | 1·16 | 0·99 to 1·37 |
| Traumatic brain injury | 1·12 | 1·08 to 1·16 | 1·31 | 1·26 to 1·36 |
| Antidepressants or depression | 1·18 | 1·16 to 1·20 | 1·27 | 1·24 to 1·31 |
| Glucocorticoids | 1·16 | 1·13 to 1·19 | 1·14 | 1·09 to 1·19 |
| Levothyroxine | 1·01 | 0·99 to 1·03 | 0·99 | 0·94 to 1·04 |
| Neuroleptics or psychosis | 1·24 | 1·21 to 1·28 | 1·42 | 1·35 to 1·48 |
| Sedatives | 1·09 | 1·08 to 1·11 | 1·17 | 1·14 to 1·20 |

CI = confidence interval. HR = hazard ratio.

Supplemental table 2. Population attributable fraction (PAF) for the different risk factors and in total

**for the outcome of hip fracture during 5 years of follow up.**

|  | **Women** | | **Men** | |
| --- | --- | --- | --- | --- |
| **Variable** | **PAF%** | **95% CI** | **PAF%** | **95% CI** |
| Age (upper half) | 79·7 | 79·3 to 80·1 | 70·3 | 69·5 to 71·0 |
| Osteoporosis | 0·5 | 0·4 to 0·5 | 0·2 | 0·2 to 0·2 |
| Parkinson's disease | 0·4 | 0·3 to 0·4 | 1·1 | 1·0 to 1·2 |
| Alcohol abuse | 0·3 | 0·3 to 0·4 | 0·9 | 0·7 to 1·1 |
| Dementia | 0·2 | 0·2 to 0·2 | 3·0 | 2·8 to 3·2 |
| Hip fracture in sibling | 0·0 | 0·0 to 0·0 | 0·0 | 0·0 to 0·0 |
| Previous fracture | 7·2 | 6·9 to 7·5 | 6·8 | 6·5 to 7·1 |
| Rheumatoid arthritis | 0·1 | 0·0 to 0·2 | 0·1 | 0·0 to 0·3 |
| Chronic obstructive disease | 0·6 | 0·5 to 0·7 | 1·1 | 0·9 to 1·2 |
| Renal disease | 0·0 | 0·0 to 0·1 | 0·2 | 0·2 to 0·3 |
| Stroke | 0·7 | 0·5 to 0·10 | 1·7 | 1·3 to 2·0 |
| Depression or antidepressants | 0·0 | 0·0 to 0·1 | 1·3 | 0·8 to 1·8 |
| Psychosis or neuroleptics | 0·4 | 0·2 to 0·6 | 0·3 | 0·0 to 0·5 |
| Swedish background | 29·6 | 27·8 to 31·4 | 37·0 | 34·6 to 39·4 |
| Lowest fifth of income | 30·4 | 28·6 to 32·2 | 36·5 | 35·3 to 37·7 |
| Nursing home resident | 7·3 | 7·0 to 7·5 | 5·4 | 5·1 to 5·8 |
| Homemaker service | 23·8 | 23·6 to 24·1 | 19·1 | 18·8 to 19·4 |
| Oral corticosteroids | 0·4 | 0·2 to 0·6 | 0·1 | to 0·1 to 0·4 |
| No diagnosis or drugs | 22·4 | 21·4 to 23·4 | 25·7 | 24·6 to 26·8 |
| **All risk factors** | **93·9** | **93·7 to 94·1** | **92·7** | **92·3 to 93·0** |

Supplementary table 3. Predicted individual 10-year risk of hip fracture in all individuals and by selected diagnoses, living conditions, medications, and income at baseline.

| **Variable** | Mean% | Median% | **Women**  25^th^ percentile | 75^th^ percentile | Mean% | Median% | **Men**  25^th^ percentile | 75^th^ percentile |
| --- | --- | --- | --- | --- | --- | --- | --- | --- |
| Osteoporosis | 12·7 | 4·3 | 1·5 | 16·6 | 9·8 | 2·8 | 1·0 | 10·7 |
| Parkinson's disease | 17·0 | 10·7 | 5·1 | 24·3 | 12·5 | 6·7 | 3·2 | 16·5 |
| Alcohol abuse | 12·1 | 5·9 | 2·5 | 16·7 | 7·7 | 3·3 | 1·5 | 9·3 |
| Dementia | 14·3 | 6·4 | 2·5 | 20·0 | 8·7 | 2·7 | 1·0 | 9·8 |
| Hip fracture in sibling | 15·3 | 4·0 | 1·2 | 19·5 | 6·0 | 1·9 | 0·7 | 6·5 |
| Previous fracture | 12·6 | 5·2 | 2·1 | 17·1 | 8·7 | 3·2 | 1·3 | 10·2 |
| Rheumatoid arthritis | 11·9 | 4·8 | 1·8 | 16·1 | 7·1 | 2·3 | 0·9 | 7·8 |
| Chronic obstructive pulmonary disease | 13·0 | 5·1 | 1·9 | 17·5 | 7·8 | 2·6 | 1·0 | 8·8 |
| Renal disease | 12·1 | 5·6 | 2·3 | 16·7 | 7·6 | 2·8 | 1·1 | 8·8 |
| Stroke | 11·5 | 4·8 | 1·9 | 15·4 | 6·9 | 2·6 | 1·1 | 8·0 |
| Depression or antidepressants | 10·9 | 3·4 | 1·1 | 13·8 | 6·6 | 1·7 | 0·6 | 6·7 |
| Psychosis or neuroleptics | 11·2 | 4·0 | 1·5 | 14·6 | 6·7 | 2·2 | 0·8 | 7·4 |
| Swedish background | 10·6 | 3·4 | 1·1 | 13·5 | 6·0 | 1·7 | 0·6 | 6·3 |
| Lowest fifth of income | 10·6 | 3·5 | 1·3 | 13·2 | 6·3 | 2·2 | 0·9 | 6·6 |
| Living in nursing home | 13·1 | 5·9 | 2·3 | 18·2 | 7·4 | 2·4 | 0·9 | 8·3 |
| Home care service | 16·4 | 10·9 | 5·2 | 23·8 | 10·8 | 6·1 | 2·9 | 14·6 |
| Oral corticosteroids | 10·9 | 3·5 | 1·2 | 13·9 | 5·9 | 1·7 | 0·6 | 6·1 |
| No diagnosis or drugs | 9·5 | 2·7 | 1·0 | 11·5 | 5·1 | 1·3 | 0·5 | 5·1 |
| All individuals | 10·4 | 3·2 | 1·1 | 13·1 | 5·9 | 1·6 | 0·6 | 6·1 |

Supplementary table 4. Validation of different thresholds for the predicted individual 10-year risk of hip fracture in the total population using the final prediction model.

**Women Men**

| Threshold % | Sensitivity | Specificity | Correctly classified% | ROC area | NNT | Sensitivity | Specificity | Correctly classified% | ROC area | NNT |
| --- | --- | --- | --- | --- | --- | --- | --- | --- | --- | --- |
| 1 | 97·9 | 24·3 | 29·1 | 61·1 | 40·6 | 93·8 | 39·1 | 40·9 | 66·4 | 64·6 |
| 2 | 94·6 | 42·1 | 45·5 | 68·3 | 32·8 | 87·9 | 55·6 | 56·7 | 71·7 | 50·9 |
| 3 | 91·7 | 51·2 | 53·8 | 71·5 | 29·0 | 83·0 | 64·0 | 64·6 | 73·5 | 44·2 |
| 4 | 89·0 | 57·3 | 59·4 | 73·2 | 26·4 | 78·7 | 69·3 | 69·6 | 74·0 | 40·1 |
| 5 | 86·4 | 61·7 | 63·3 | 74·1 | 24·7 | 74·7 | 73·3 | 73·3 | 74·0 | 37·0 |
| 6 | 83·9 | 65·1 | 66·3 | 74·5 | 23·4 | 71·0 | 76·3 | 76·1 | 73·6 | 34·8 |
| 7 | 81·4 | 67·8 | 68·7 | 74·6 | 22·4 | 67·3 | 78·7 | 78·3 | 73·0 | 33·1 |
| 8 | 79·0 | 70·0 | 70·6 | 74·5 | 21·6 | 63·8 | 80·8 | 80·2 | 72·3 | 31·7 |
| 9 | 76·6 | 72·1 | 72·4 | 74·3 | 20·9 | 60·4 | 82·5 | 81·7 | 71·4 | 30·7 |
| 10 | 74·3 | 73·8 | 73·8 | 74·0 | 20·3 | 57·1 | 84·0 | 83·1 | 70·5 | 29·7 |
| 11 | 72·0 | 75·2 | 75·0 | 73·6 | 19·9 | 54·0 | 85·3 | 84·2 | 69·6 | 29·0 |
| 12 | 69·7 | 76·6 | 76·2 | 73·2 | 19·5 | 51·1 | 86·4 | 85·2 | 68·8 | 28·3 |
| 13 | 67·5 | 77·8 | 77·2 | 72·7 | 19·1 | 48·4 | 87·5 | 86·1 | 67·9 | 27·8 |
| 14 | 65·4 | 78·9 | 78·1 | 72·2 | 18·8 | 45·7 | 88·4 | 86·9 | 67·0 | 27·3 |
| 15 | 63·3 | 79·9 | 78·9 | 71·6 | 18·6 | 43·2 | 89·2 | 87·6 | 66·2 | 26·9 |

The model included the 19 predictors presented in supplementary table 2.

NNT = number needed to treat to prevent one hip fracture assuming a treatment effect of 30%.

Supplementary table 5. Descriptive characteristics of the cohort with non-Swedish background (N=504 431).

|  | **Women** |  | **Men** |  |
| --- | --- | --- | --- | --- |
| **Variable** | **(N=267 982)** |  | **(N=236 449)** |  |
| Age, years ± SD | 63·8 ± 10·4 |  | 61·8 ± 9·1 |  |
| Income, Euro ± SD | 12 292 ± 12 106 |  | 15 850 ± 29 067 |  |
| Hip fracture in full sibling, N % | 110 | 0·0 | 134 | 0·0 |
| Fracture in full sibling, N % | 1518 | 0·3 | 1615 | 0·3 |
| Nursing home resident, N % | 5310 | 2·0 | 2317 | 1·0 |
| Homemaker service, N %  **Diagnoses and medications, N %** | 13 592 | 5·2 | 6225 | 2·6 |
| Alcohol abuse | 2205 | 0·4 | 4851 | 1·0 |
| Angina pectoris | 11 729 | 2·2 | 15 156 | 3·2 |
| Any fracture | 21 594 | 4·0 | 10 425 | 2·2 |
| Asthma | 5601 | 1·0 | 2795 | 0·6 |
| Atrial fibrillation | 7011 | 1·3 | 7683 | 1·6 |
| Bipolar disease | 663 | 0·1 | 401 | 0·1 |
| Cancer | 33 062 | 6·0 | 16 085 | 3·3 |
| Crohn’s disease | 703 | 0·1 | 591 | 0·1 |
| Chronic obstructive pulmonary disease | 3964 | 0·7 | 3582 | 0·7 |
| Colitis | 950 | 0·2 | 1072 | 0·2 |
| Dementia | 3295 | 0·6 | 2524 | 0·5 |
| Diabetes mellitus | 19 788 | 3·8 | 22 329 | 4·8 |
| Myocardial infarction | 4794 | 0·9 | 8786 | 1·8 |
| Osteoporosis | 2538 | 0·5 | 322 | 0·1 |
| Parkinson's disease | 655 | 0·1 | 711 | 0·1 |
| Renal disease | 363 | 0·1 | 543 | 0·1 |
| Rheumatoid arthritis | 3456 | 0·6 | 1164 | 0·2 |
| Stroke | 5806 | 1·1 | 6193 | 1·3 |
| Thyrotoxicosis | 2492 | 0·5 | 567 | 0·1 |
| Traumatic brain injury | 7033 | 1·3 | 9016 | 1·9 |
| Antidepressants or depression | 34 866 | 6·5 | 16 809 | 3·6 |
| Bisphosphonates | 6328 | 1·1 | 704 | 0·1 |
| Glucocorticoids | 10 686 | 2·0 | 5967 | 1·2 |
| Levothyroxine | 25 895 | 4·8 | 4270 | 0·9 |
| Neuroleptics or psychosis | 7 720 | 1·4 | 4656 | 1·0 |
| Sedatives | 38 926 | 7·2 | 19 348 | 4·1 |

Supplementary table 6. Independent risk factors for 5-year risk of hip fractures in individuals with non-Swedish background (N=497 399).

| **Variables** | **Women (N=261 654)**  HR | 95% CI | **Men (N=235 745)**  HR | 95% CI |
| --- | --- | --- | --- | --- |
| Age per year to increase | 1·11 | 1·10 to 1·11 | 1·10 | 1·10 to 1·11 |
| Income per €1000 to increase | 0·97 | 0·93 to 1·01 | 0·84 | 0·79 to 0·89 |
| Hip fracture in full sibling | 3·65 | 0·97 to 13·77 | 0·91 | 0·12 to 7·09 |
| Fracture in full sibling | 0·97 | 0·48 to 1·94 | 1·52 | 0·82 to 2·84 |
| Nursing home resident | 1·35 | 1·23 to 1·48 | 1·53 | 1·30 to 1·80 |
| Homemaker service  **Diagnoses and medications** | 1·89 | 1·77 to 2·03 | 2·11 | 1·86 to 2·38 |
| Alcohol abuse | 3·06 | 2·46 to 3·81 | 2·81 | 2·33 to 3·39 |
| Angina pectoris | 1·03 | 0·93 to 1·14 | 0·83 | 0·71 to 0·96 |
| Any fracture | 1·55 | 1·44 to 1·66 | 2·44 | 2·17 to 2·76 |
| Asthma | 0·95 | 0·81 to 1·12 | 0·81 | 0·59 to 1·11 |
| Atrial fibrillation | 1·25 | 1·13 to 1·38 | 1·12 | 0·96 to 1·31 |
| Bipolar disease | 0·97 | 0·58 to 1·63 | 1·65 | 0·84 to 3·22 |
| Cancer | 1·20 | 1·12 to 1·29 | 1·33 | 1·18 to 1·49 |
| Crohn’s disease | 1·77 | 1·12 to 2·80 | 1·52 | 0·75 to 3·07 |
| Chronic obstructive pulmonary disease | 1·78 | 1·54 to 2·05 | 1·83 | 1·50 to 2·22 |
| Colitis | 1·23 | 0·80 to 1·88 | 1·05 | 0·56 to 1·98 |
| Dementia | 1·47 | 1·31 to 1·66 | 1·37 | 1·12 to 1·67 |
| Diabetes mellitus | 1·24 | 1·14 to 1·35 | 1·35 | 1·20 to 1·51 |
| Myocardial infarction | 1·09 | 0·95 to 1·25 | 1·10 | 0·92 to 1·32 |
| Osteoporosis | 1·21 | 0·97 to 1·51 | 1·62 | 0·73 to 3·62 |
| Parkinson's disease | 1·70 | 1·31 to 2·22 | 2·65 | 2·00 to 3·51 |
| Renal disease | 1·06 | 0·64 to 1·77 | 1·04 | 0·60 to 1·79 |
| Rheumatoid arthritis | 1·33 | 1·08 to 1·64 | 2·05 | 1·40 to 3·00 |
| Stroke | 1·31 | 1·17 to 1·46 | 1·45 | 1·25 to 1·68 |
| Thyrotoxicosis | 0·98 | 0·74 to 1·30 | 1·10 | 0·51 to 2·33 |
| Traumatic brain injury | 1·24 | 1·09 to 1·42 | 1·38 | 1·17 to 1·63 |
| Antidepressants or depression | 1·22 | 1·14 to 1·32 | 1·25 | 1·10 to 1·43 |
| Glucocorticoids | 1·32 | 1·17 to 1·49 | 1·17 | 0·94 to 1·45 |
| Levothyroxine | 1·04 | 0·95 to 1·13 | 1·06 | 0·82 to 1·37 |
| Neuroleptics or psychosis | 1·34 | 1·20 to 1·50 | 1·35 | 1·10 to 1·65 |
| Sedatives | 1·19 | 1·11 to 1·27 | 1·17 | 1·04 to 1·31 |

CI = confidence interval. HR = hazard ratio.

Supplementary table 7. Predicted individual 5-year risk of hip fracture in all 497 399 individuals with non-Swedish background and by selected diagnoses, living conditions, medications, and income at baseline.

Women Men

| **Variable** | Mean% | Median% | 25^th^ percentile | 75th percentile | Mean% | Median% | 25^th^ percentile | 75^th^ percentile |
| --- | --- | --- | --- | --- | --- | --- | --- | --- |
| Osteoporosis | 2·7 | 0·6 | 0·3 | 2·3 | 1·5 | 0·3 | 0·1 | 0·9 |
| Parkinson's disease | 3·7 | 2·0 | 1·1 | 4·3 | 2·8 | 1·1 | 0·5 | 2·9 |
| Alcohol abuse | 3·7 | 1·4 | 0·7 | 3·8 | 1·9 | 0·9 | 0·5 | 2·0 |
| Dementia | 3·1 | 0·9 | 0·4 | 2·9 | 1·4 | 0·3 | 0·1 | 1·1 |
| Previous fracture | 3·2 | 1·2 | 0·6 | 3·2 | 2·1 | 0·9 | 0·5 | 2·2 |
| Rheumatoid arthritis | 2·5 | 1·0 | 0·5 | 2·6 | 2·0 | 0·5 | 0·2 | 1·6 |
| Chronic obstructive pulmonary disease | 3·5 | 1·2 | 0·5 | 3·4 | 1·7 | 0·5 | 0·2 | 1·5 |
| Renal disease | 2·5 | 1·0 | 0·5 | 2·6 | 1·2 | 0·4 | 0·2 | 1·2 |
| Stroke | 2·9 | 1·0 | 0·4 | 2·8 | 1·5 | 0·7 | 0·3 | 1·5 |
| Depression or antidepressants | 2·5 | 0·6 | 0·3 | 2·2 | 1·3 | 0·4 | 0·1 | 1·1 |
| Psychosis or neuroleptics | 2·6 | 0·6 | 0·3 | 2·2 | 1·3 | 0·5 | 0·2 | 1·3 |
| Lowest fifth of income | 2·4 | 0·6 | 0·3 | 2·0 | 1·2 | 0·4 | 0·2 | 1·1 |
| Nursing home resident | 3·9 | 1·5 | 0·7 | 4·1 | 1·8 | 0·6 | 0·2 | 1·6 |
| Homemaker service | 4·4 | 2·3 | 1·1 | 5·4 | 2·6 | 1·4 | 0·7 | 3·1 |
| Oral corticosteroids | 2·7 | 0·7 | 0·3 | 2·3 | 1·2 | 0·5 | 0·3 | 1·2 |
| No diagnosis or drugs | 1·8 | 0·5 | 0·2 | 1·6 | 0·9 | 0·3 | 0·1 | 0·8 |
| All individuals | 2·7 | 0·6 | 0·3 | 2·3 | 1·1 | 0·3 | 0·1 | 1·0 |

Supplementary table 8. Validation of different thresholds for the predicted individual 5-year risk of hip fracture using the final prediction model in individuals with non-Swedish background.

Women Men

| Threshold % | Sensitivity | Specificity | Correctly classified% | ROC to area | NNT | Sensitivity | Specificity | Correctly classified% | | ROC to area | NNT |
| --- | --- | --- | --- | --- | --- | --- | --- | --- | --- | --- | --- |
| 1 | 90·2 | 64·0 | 64·5 | 77·1 | 70·9 | 77·4 | 75·8 | 75·9 | 76·6 | | 113·2 |
| 2 | 82·1 | 76·7 | 76·8 | 79·4 | 51·5 | 63·4 | 87·2 | 87·0 | 75·3 | | 74·3 |
| 3 | 74·6 | 82·7 | 82·5 | 78·7 | 42·6 | 52·3 | 91·7 | 91·3 | 72·0 | | 59·3 |
| 4 | 68·0 | 86·4 | 86·0 | 77·2 | 37·3 | 44·1 | 94·2 | 93·7 | 69·1 | | 49·7 |
| 5 | 62·0 | 88·7 | 88·2 | 75·4 | 34·1 | 37·9 | 95·7 | 95·2 | 66·8 | | 43·3 |
| 6 | 57·6 | 90·5 | 89·9 | 74·1 | 31·2 | 32·0 | 96·6 | 96·0 | 64·3 | | 40·2 |
| 7 | 53·2 | 91·9 | 91·1 | 72·5 | 29·2 | 28·0 | 97·3 | 96·7 | 62·6 | | 36·9 |
| 8 | 48·8 | 93·0 | 92·1 | 70·9 | 27·8 | 23·6 | 97·9 | 97·2 | 60·7 | | 35·1 |
| 9 | 45·4 | 93·8 | 92·9 | 69·6 | 26·4 | 20·6 | 98·2 | 97·5 | 59·4 | | 33·5 |
| 10 | 42·1 | 94·6 | 93·5 | 68·3 | 25·2 | 17·6 | 98·5 | 97·8 | 58·1 | | 32·6 |

The model included 17 of the predictors presented in supplementary table 2 (hip fracture in sibling was excluded because of the poor linkage to relatives in those with non-Swedish background). NNT = number needed to treat to prevent one hip fracture assuming a treatment effect of 30%.

Supplementary figure 1. Individual standardized 10-year risk of hip fracture according to age at baseline in all women and by different risk factors.


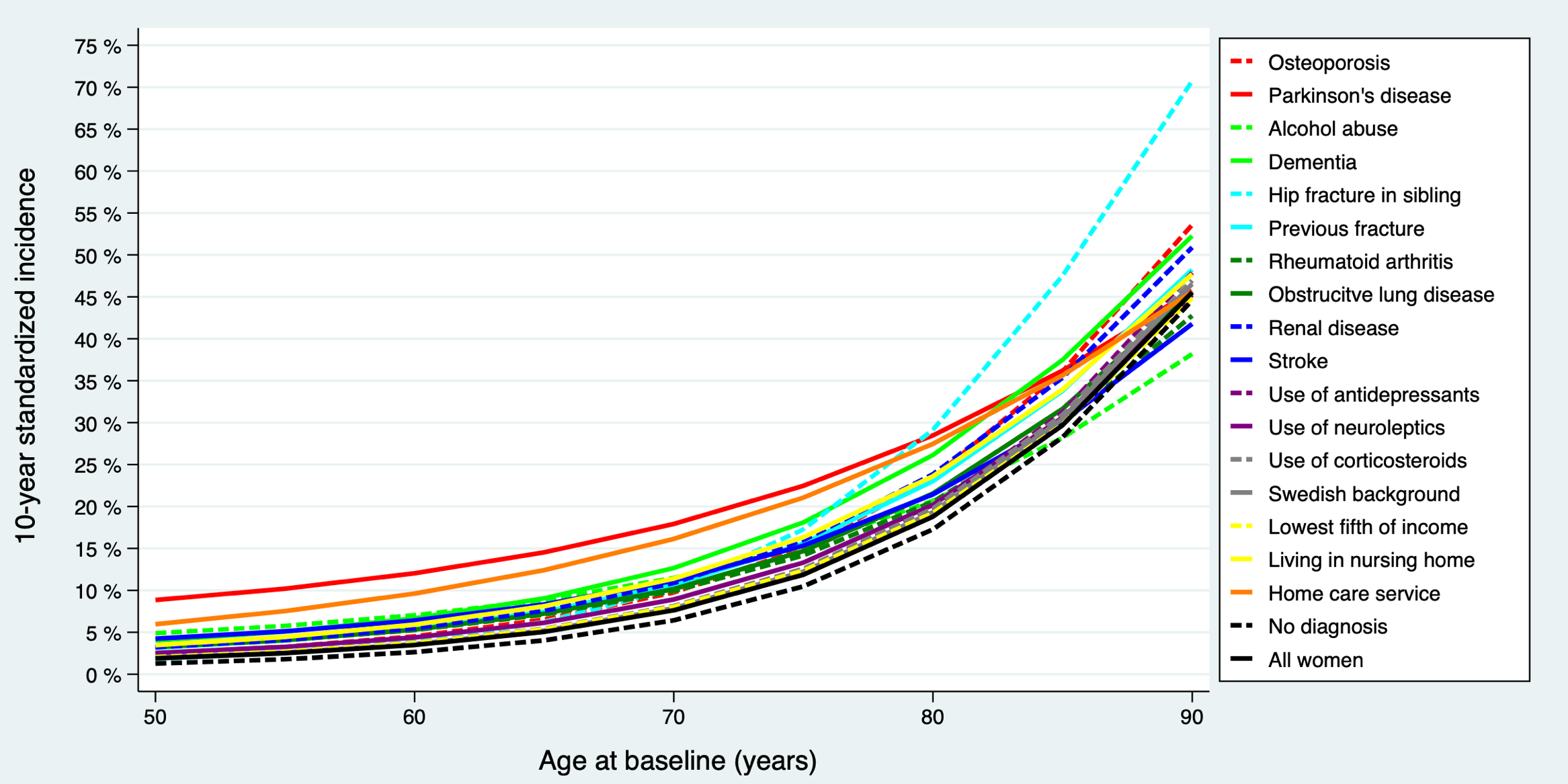


Supplementary figure 2. Individual standardized 10-year risk of hip fracture according to age at baseline in all men and by different risk factors.


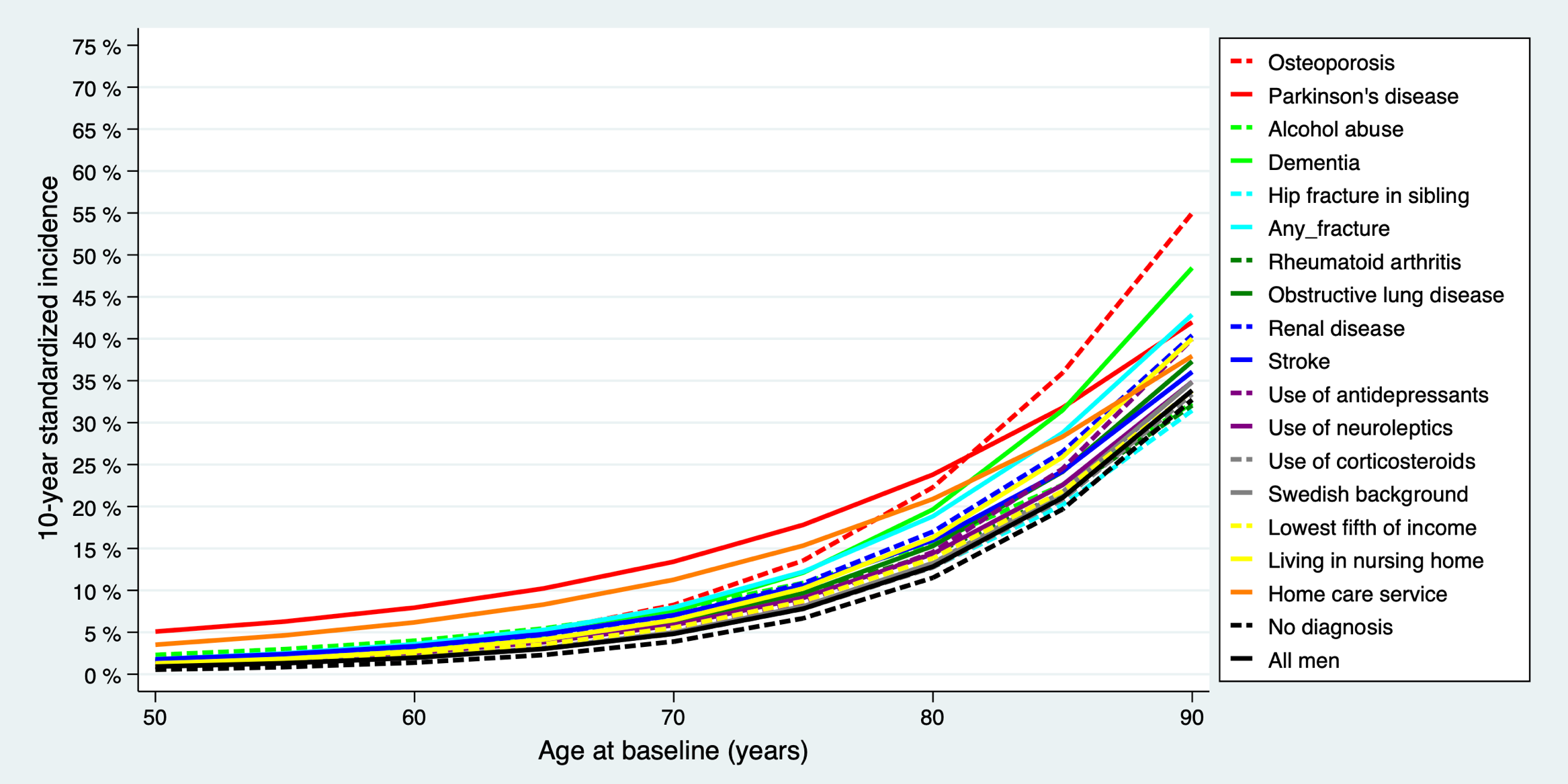


Supplementary figure 3. Calibration plot in women showing the observed risk of hip fracture on the x-axis and the predicted risk of hip fracture from the final model on the y-axis during 5 years of follow up.

Supplementary figure 4. Calibration plot in men showing the observed risk of hip fracture on the x-axis and the predicted risk of hip fracture from the final model on the y-axis during 5 years of follow up.

Supplementary figure 5. Individual standardized 10-year risk of hip fracture according to age at baseline in all women with non to Swedish background and by different risk factors.


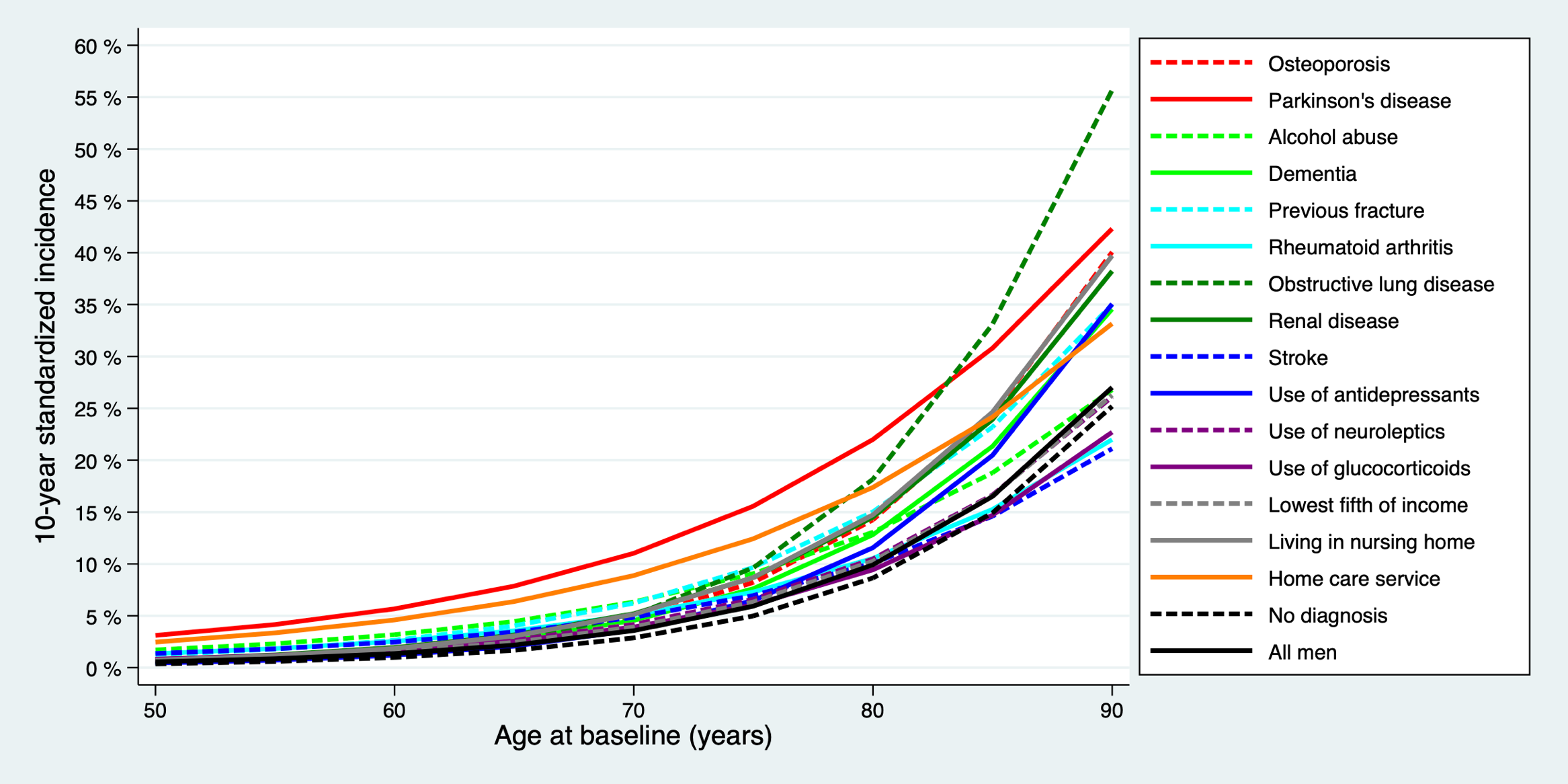


Supplementary figure 6. Individual standardized 10-year risk of hip fracture according to age at baseline in all men with non to Swedish background and by different risk factors.

**
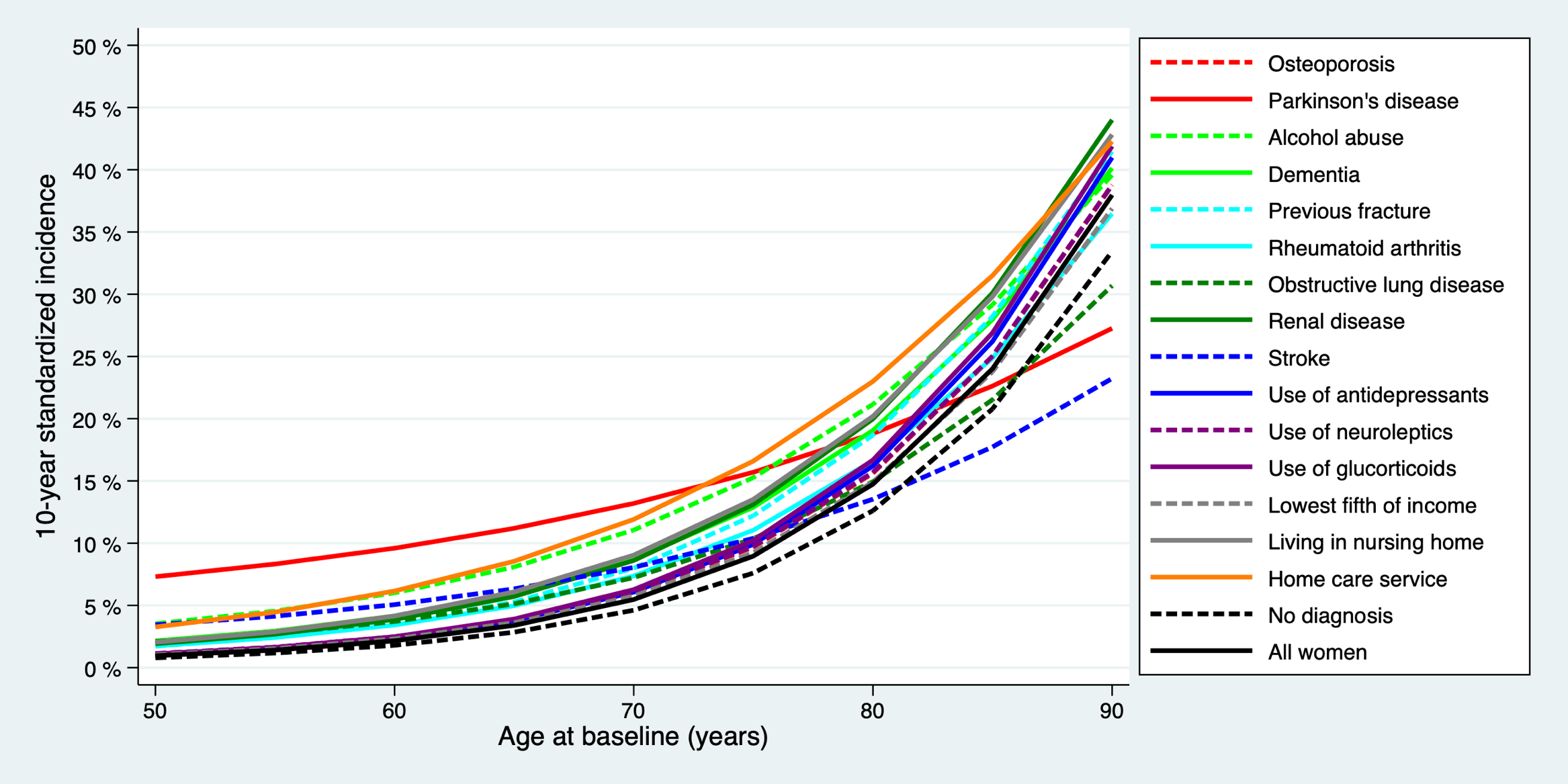
**
